# Troxerutin Prevents 5-Fluorouracil Induced Morphological Changes in the Intestinal Mucosa: Role of Cyclooxygenase-2 Pathway

**DOI:** 10.3390/ph13010010

**Published:** 2020-01-08

**Authors:** João Antônio Leal de Miranda, Conceição da Silva Martins, Lázaro de Sousa Fideles, Maria Lucianny Lima Barbosa, João Erivan Façanha Barreto, Helder Bindá Pimenta, Francisco Orlando Rafael Freitas, Paulo Vitor de Souza Pimentel, Claudio Silva Teixeira, Ariel Gustavo Scafuri, Maria Claudia dos Santos Luciano, Joabe Lima Araújo, Jefferson Almeida Rocha, Icaro Gusmão Pinto Vieira, Nágila Maria Pontes Silva Ricardo, Matheus da Silva Campelo, Maria Elenir Nobre Pinho Ribeiro, Gerly Anne de Castro Brito, Gilberto Santos Cerqueira

**Affiliations:** 1 Department of Morphology, Faculty of Medicine, Federal University of Ceará, s/n Delmiro of Farias Street, Porangabuçu Campus, Fortaleza 60416-030, Brazil; josycristophe@hotmail.com (C.d.S.M.); lazarofideles@gmail.com (L.d.S.F.); marialucianny@gmail.com (M.L.L.B.); erivanfacanha@yahoo.com.br (J.E.F.B.); hbinda@uea.edu.br (H.B.P.); francisco.orlando@gmail.com (F.O.R.F.); paulo_vitordesouza@hotmail.com (P.V.d.S.P.); claudioanatomia@yahoo.com.br (C.S.T.); urologia@gmail.com (A.G.S.); gerlybrito@hotmail.com (G.A.d.C.B.); giufarmacia@hotmail.com (G.S.C.); 2 Nucleus of Research and Development of Medications (NPDM), Federal University of Ceará, Coronel Nunes of Melo Street, 100, Fortaleza 60430-275, Brazil; claudia_santos_luciano@hotmail.com; 3 Research Group in Natural Sciences and Biotechnology, Federal University of Maranhão, s/n Avenue Aurila Maria Santos Barros of Sousa, Frei Alberto Beretta, Grajaú-MA 65940-000, Brazil; joabearaujobiotec@gmail.com (J.L.A.); jeffersonkalel@hotmail.com (J.A.R.); 4 Technological Development Park, Federal University of Ceará, Humberto Monte Avenue, 2977, Pici Campus, Fortaleza 60440-900, Brazil; icarogpv@uol.com.br; 5 Department of Organic and Inorganic Chemistry, Federal University of Ceará, Pici Campus, Fortaleza 60440-900, Brazil; naricard@ufc.br (N.M.P.S.R.); matheus.campelo@hotmail.com (M.d.S.C.); elenir.ribeiro@ufc.br (M.E.N.P.R.)

**Keywords:** chemoprevention, inflammation, flavonoid, intestine

## Abstract

Intestinal mucositis is a common complication associated with 5-fluorouracil (5-FU), a chemotherapeutic agent used for cancer treatment. Troxerutin (TRX), a semi-synthetic flavonoid extracted from *Dimorphandra gardneriana*, has been reported as a potent antioxidant and anti-inflammatory agent. In the present study, we aimed to evaluate the effect of TRX on 5-FU-induced intestinal mucositis. Swiss mice were randomly divided into seven groups: Saline, 5-FU, TRX-50, TRX-100, TRX-150, Celecoxib (CLX), and CLX + TRX-100. The weight of mice was measured daily. After treatment, the animals were euthanized and segments of the small intestine were collected to evaluate histopathological alterations (morphometric analysis), levels of malondialdehyde (MDA), myeloperoxidase (MPO), glutathione (GSH), mast and goblet cell counts, immunohistochemical analysis, and cyclooxygenase-2 (COX-2) activity. Compared to the saline treatment, the 5-FU treatment induced intense weight loss and reduction in villus height. TRX treatment (100 mg/kg) prevented the 5-FU-induced histopathological changes and decreased oxidative stress by decreasing the MDA levels and increasing GSH concentration. TRX attenuated inflammatory process by decreasing MPO activity, intestinal mastocytosis, and COX-2 expression. TRX also reversed the depletion of goblet cells. Our findings suggest that TRX at a concentration of 100 mg/kg had chemopreventive effects on 5-FU-induced intestinal mucositis via COX-2 pathway.

## 1. Introduction

Oral and gastrointestinal mucositis are the most common adverse effects of cancer chemotherapy [1,2]. Intestinal mucositis, characterized by inflammatory and/or ulcerative process in the gastrointestinal tract, is a result of cellular and tissue damage caused by 5-fluorouracil (5-FU) antimetabolite treatment [3]. Based on the number of chemotherapy cycles, 5-FU dose, schedule, patient’s age, sex, and nutritional status [4,5], the incidences of mucositis in cancer patients range from 40 to 100% [6,7,8].

The pathophysiology of mucositis is more complex than mere direct damage to the intestinal epithelium. It includes sequential interaction of events, which leads to generation of reactive oxygen and nitrogen species (ROS/RNS). Nuclear factor kappa B (NF-kB), proinflammatory cytokines, such as interleukin 1 beta (IL-1β), interleukin 6 (IL-6), and tumor necrosis factor alpha (TNF-α), and apoptotic mediators have been known to be associated with this process. The interaction of these factors results in oxidative damage, intense infiltration of inflammatory cells, villous atrophy, crypt hypoplasia, edema, necrosis, and cell death, which ultimately leads to damage and rupture of the intestinal epithelial barrier [4,9,10,11,12,13,14].

Due to the lack of effective therapeutic tools for the treatment of intestinal mucositis, new alternative treatments that can reduce the side effects of 5-FU without affecting the cancer treatment have been a field of active research. In this regard, natural products, such as flavonoids are considered as an important source because of their better compatibility with the human system, few side effects, and various pharmacological activities [15,16].

Troxerutin (TRX) is a semi-synthetic flavonoid, derived from rutin, which is extracted from *Dimorphandra gardneriana*. This compound has various pharmacological activities, including antioxidant [17,18,19], anti-inflammatory [20], anti-apoptotic [21,22], and antiedematogenic [23,24]. Further, it is a potent therapeutic agent for neuropathic pain [25]. The aim of the current study was to determine the preventive effects of TRX on 5-FU-induced intestinal mucositis and explore the possible mechanisms of action involved.

## 2. Results

### 2.1. Characterization of Troxerutin

The synthesized TRX had a retention time of 4.43 min and a purity of 98.57% based on HPLC analysis. The results corroborate with those reported by Satinsky et al. [26], indicating that the semi-synthesis process employed provided TRX as a majoritarian product.

**Figure 1:**
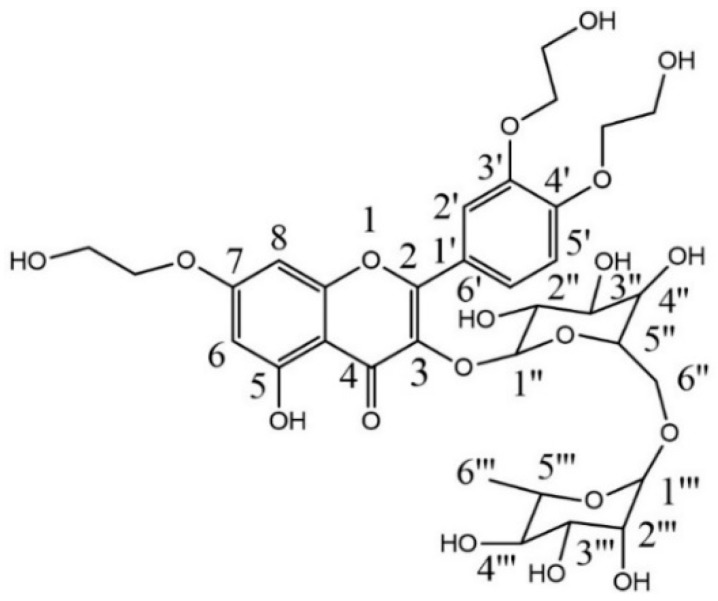
Chemical structure of troxerutin.

The DEPT-135 spectrum signals between δ 70.9 and δ 59.7 were observed for the presence of ether oxygenated methylene groups (OCH_2_CH_2_OH). The data indicated a mix of O-β-hydroxyethylated compounds, where the majoritarian compound was trihydroxyethylrutin (Figure 1). Structural elucidation of TRX, semi-synthesized by ^1^H and ^13^C NMR are shown in Table 1. Such attributions corroborate with those reported by Xiao et al. [27] and Xu et al. [28].

### 2.2. Weight Analysis

**Figure 2:**
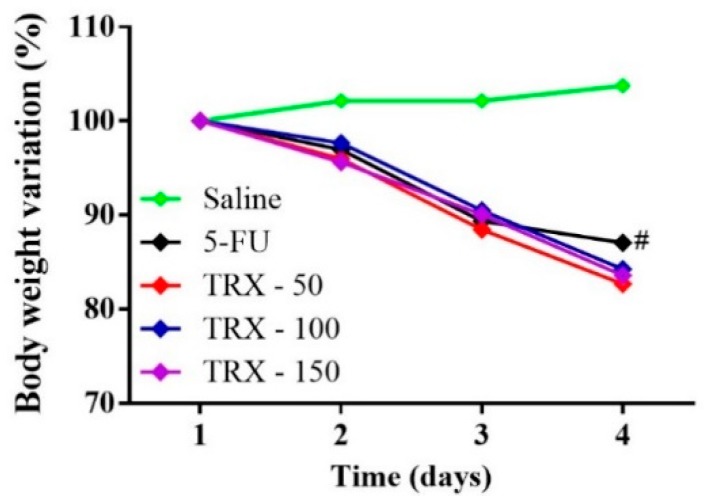
Body weight variation in mice subjected to induced intestinal mucositis (5-FU, 450 mg/kg, ip, single dose) and treated with TRX (50, 100, and 150 mg/kg for 3 days). The results are expressed as the mean ± SEM of the weight evaluation percentage of the initial weight, of a minimum of six animals per group. Two-way ANOVA followed by the Tukey’s test were used for the statistical analysis, where # *p* < 0.05 vs. saline and * *p* < 0.05 vs. 5-FU.

Weight loss is one of the most common side effects of 5-FU chemotherapy treatment. The experimental mice models induced with intestinal mucositis using 5-FU showed a significant loss of weight. As shown in Figure 2, from the second day, all mice submitted to 5-FU-induced intestinal mucositis had progressive weight loss, which was significant compared to the saline group (*p* < 0.05). However, the pretreatment with TRX at any dose did not prevent the weight loss in the animals with 5-FU-induced mucositis (*p* < 0.05).

### 2.3. Histopathological and Morphometric Analysis

As shown in Table 2, administration of 5-FU induced changes in the intestinal mucosa of mice, as evidenced from reduced villus height, crypt necrosis and hypoplasia, intense inflammatory cell infiltration, vacuolization, and edema of intestinal mucosal and muscular layer cells. This resulted in a significant increase in the microscopic score in 5-FU administered group compared to the saline group (*p* < 0.05), especially in the duodenal segment. Notably, the TRX treatment (100 mg/kg) resulted in a significant decrease (*p* < 0.05) in the histopathological scores when compared to the 5-FU lesion group.

**Figure 3:**
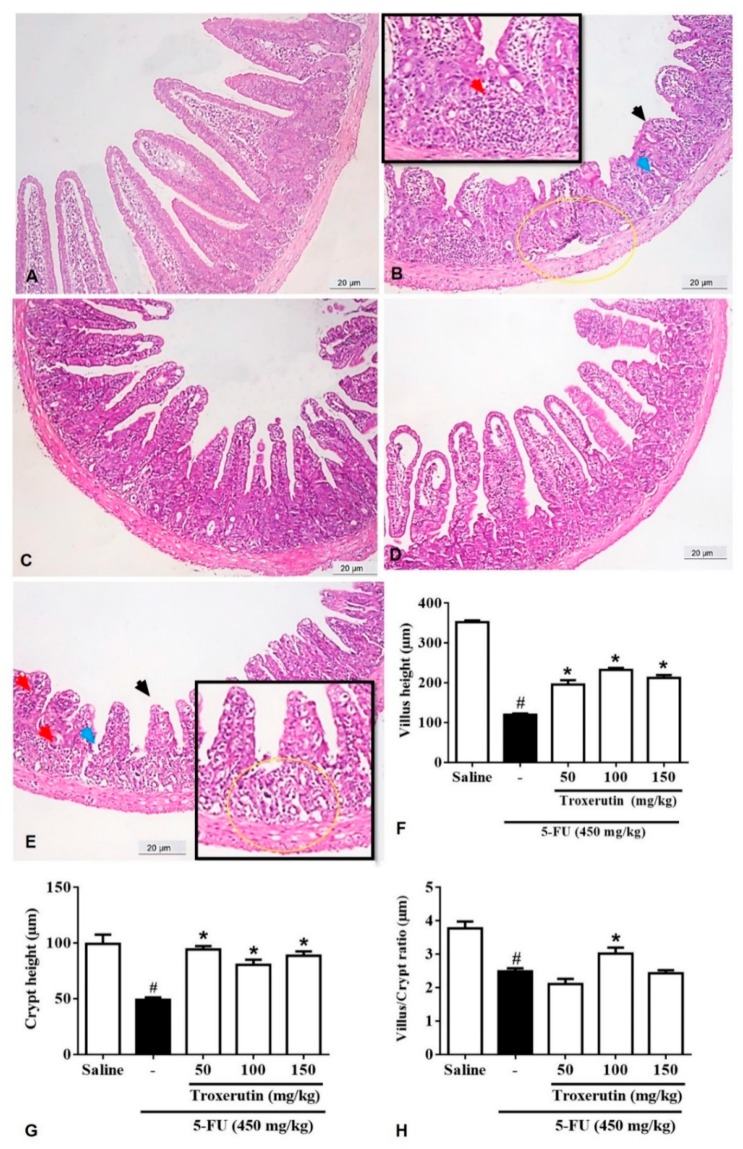
Histopathological analysis of duodenum. (**A**) Saline; (**B**) 5-FU; (**C**) TRX-50; (**D**) TRX-100; (**E**) TRX-150. 5-FU induced inflammatory cell infiltrate (red arrow), decreased intestinal villi (black arrow), loss of intestinal crypt architecture (blue arrow), edema (yellow circle). Pretreatment with TRX (50, 100 and 150 mg/kg) decreased the inflammatory infiltrate and prevented the shortening of the villi (**F**), increased crypt depth (**G**) and decreased villus/crypt ratio (**H**), with greater reversion of the 5-FU effect in the TRX 100 + 5-FU group. Values were expressed as mean ± SEM. One-way ANOVA followed by the Tukey’s test were used for the statistical analysis was used, where # *p* < 0.05 vs saline group and * *p* < 0.05 vs group 5-FU.

As shown by the morphometric analysis of duodenum (Figure 3), 5-FU treatment (Figure 3B) significantly (*p* < 0.05) shortened the villi (120.30 ± 2.90), decreased crypt depth (49.23 ± 2.01), and reduced villus/crypt ratio (2.48 ± 0.09) compared to that of the saline treatment (villus height: 352.70 ± 4.25; crypt depth: 99.30 ± 8.29; villus/crypt ratio: 3.77 ± 0.20, Figure 3A). Treatment with TRX significantly (*p* < 0.05) reversed villi shortening (50 mg/kg: 195.80 ± 11.12, Figure 3C; 100 mg/kg: 232.70 ± 4.74, Figure 3D; 150 mg/kg: 212.4 ± 7.21, Figure 3E), induced by 5-FU treatment, Figure 3F. Similarly, 5-FU-induced reduction in crypt depth was significantly reversed (*p* < 0.05, Figure 3G) by all TRX treatments (50 mg/kg: 94.36 ± 2.97; 100 mg/kg: 80.53 ± 4.57; 150 mg/kg: 88.75 ± 3.79). However, significant increase in the villus/crypt ratio was observed only in TRX-100 group (3.02 ± 0.17, *p* < 0.05) compared to that in the 5-FU group (Figure 3H).

### 2.4. Leukocyte Count

**Figure 4:**
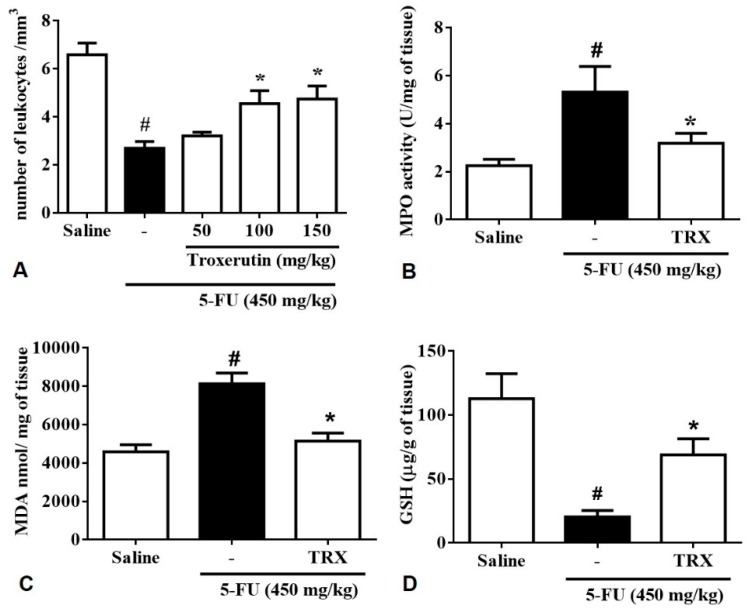
(**A**) Total leukocyte count; (**B**) Activity of myeloperoxidase (MPO); (**C**) Level of malondialdehyde (MDA); (**D**) concentration of glutathione (GSH). For evaluations of MPO, MDA, and GSH, the dose of TRX used 100 mg/kg. Values were presented as mean ± SEM. For the statistical analysis, one-way ANOVA followed by Tukey’s test was used, where # *p* < 0.05 vs saline group and * *p* < 0.05 vs group 5-FU.

As expected the analysis of leukocyte count in blood of 5-FU group (2.69 ± 0.27, *p* < 0,05) showed a significant decrease in the number of total leukocytes when (compared to that in the saline group (6.58 ± 0.48, *p* < 0.05). In contrast, TRX-100 (4.55 ± 0.53, *p* < 0.05) and TRX-150 (4.74 ± 0.54, *p* < 0.05) pretreatment prevented 5-FU-induced leukopenia (*p* < 0.05) (Figure 4A).

### 2.5. Myeloperoxidase Assay (MPO)

To investigate the effects of TRX pretreatment on neutrophil recruitment in 5-FU-induced intestinal mucositis, we determined the activity of myeloperoxidase (MPO) in the duodenum, a neutrophil marker. The 5-FU group (5.31 ± 1.06, *p* < 0.05) presented a significant increase in MPO levels in the duodenum compared to the saline group (2.24 ± 0.26, *p* < 0.05). The TRX-100 group (3.18 ± 0.41, *p* < 0.05) did not presented alterations in MPO levels compared to the negative control, however when compared with the 5-FU group the MPO levels had a significative decrease of MPO levels (Figure 4B).

### 2.6. Malondialdehyde (MDA) and Glutathione (GSH) levels

To investigate the effects of TRX pretreatment on 5-FU-induced oxidative stress in the duodenum, MDA, and GSH levels (final products of oxidative stress) were evaluated. We found that 5-FU treatment increased MDA levels (8127 ± 561.5, *p* < 0.05) in the duodenum compared to that in saline treatment (4587 ± 367.5, *p* < 0.05, Figure 4C). Administration of 100 mg/kg of TRX, significantly reduced the MDA levels (5149 ± 415.4, *p* < 0.05) compared to that of 5-FU treatment. Animals treated with 5-FU showed a significant decrease in GSH levels (20.48 ± 4.89, *p* < 0.05) compared to that in the saline group (112.8 ± 19.45, *p* < 0.05). However, the GSH levels were found to be significantly increased in the TRX-100 group (68.94 ± 12.46, *p* < 0.05) compared to that in the 5-FU group (Figure 4D).

### 2.7. Cell Count of the Intestinal Mucosa: Mast and Goblet Cells

**Figure 5:**
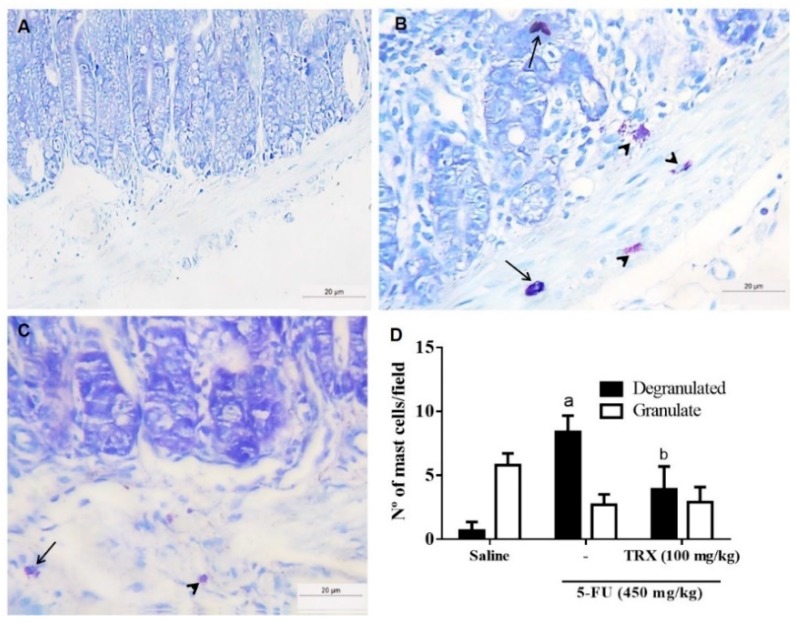
Mast cell counts in the duodenal segments based on toluidine blue staining. Panels (**A**), (**B**), and (**C**) correspond to Saline, 5-FU, and TRX-100 groups, respectively. (**D**): Statistical representation of experimental groups. Degranulated mast cells (arrow) and granulated mast cells (arrowhead). All the panels were obtained at ×400 magnification. Values are presented as mean ± SEM of the number of mast cells per field. For the statistical analysis, one-way ANOVA followed by Tukey’s test was used. a: *p* < 0.05 in 5-FU vs. Saline group; b: *p* < 0.05 in TRX-100 vs. 5-FU group.

To evaluate the effect of TRX pretreatment on 5-FU-induced mastocytosis, the number of mast cells in the duodenum was measured (Figure 5). The 5-FU treatment significantly increased the number of degranulated mast cells per field (8.40 ± 0.52, *p* < 0.05) compared to that of saline treatment (0.70 ± 0.25, *p* < 0.05). Administration of 100 mg/kg of TRX (Figure 5C) reduced the number of degranulated mast cells (3.90 ± 0.52, *p* < 0.05), thus preventing 5-FU-induced mastocytosis and degranulation in mouse intestine (Figure 5B,D).

**Figure 6:**
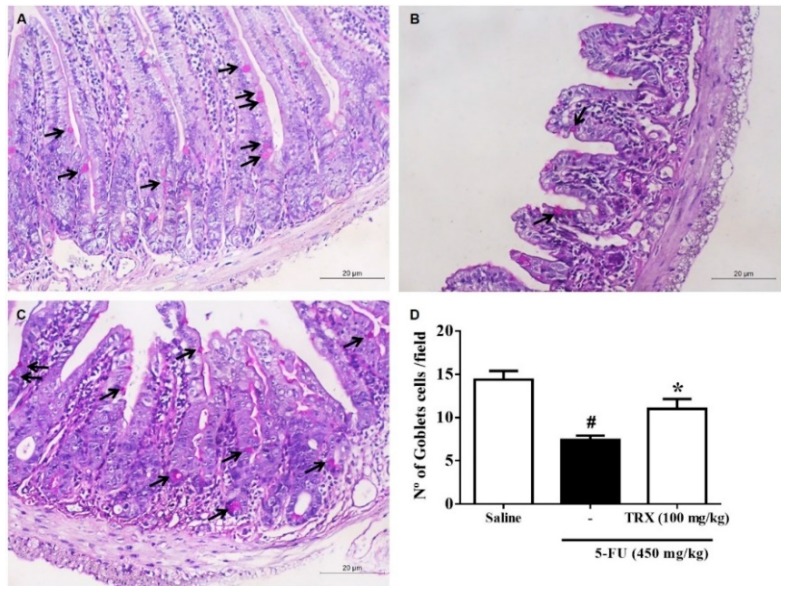
Goblet cell counts in the duodenal segment based on the Schiff periodic acid (PAS) staining. Panels (**A**), (**B**), and (**C**) correspond to Saline, 5-FU, and TRX-100 groups, respectively. (**D**): Statistical representation of experimental groups. Values are presented as mean ± SEM of the number of goblet cells per field. For the statistical analysis, one-way ANOVA followed by Tukey’s test was used. #: *p* < 0.05 in 5-FU vs. Saline group; *: *p* < 0.05 in TRX-100 vs. 5-FU group.

Analysis of goblet cells in the duodenal segment (Figure 6) showed that 5-FU treatment significantly decreased (*p* < 0.05) the number of goblet cells in the intestinal mucosa (7.42 ± 0.48) compared to that of saline treatment (14.38 ± 1.01). However, treatment with 100 mg/kg of TRX resulted in preservation of the number of goblet cells (11.00 ± 1.15, *p* < 0.05, Figure 6C) compared to that of 5-FU treatment (Figure 6B,D).

### 2.8. Effect of TRX on Cyclooxygenase-2 Pathway Based on Histopathological and Morphometric Analyses

**Figure 7:**
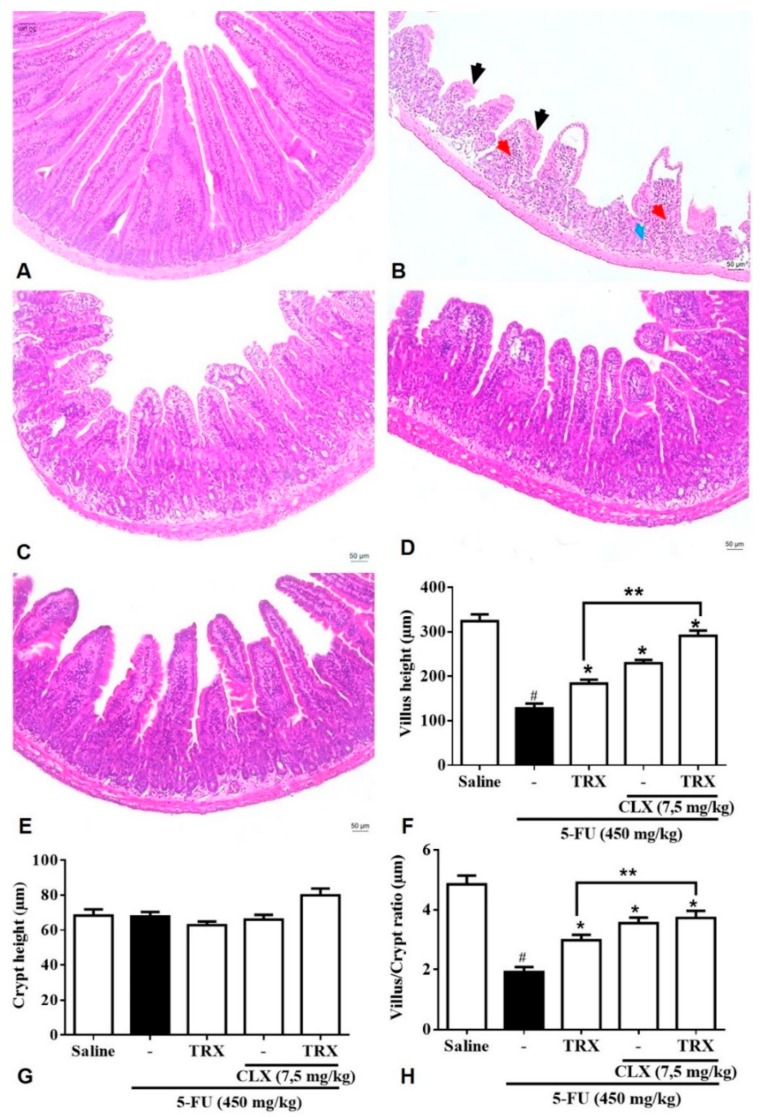
Effect of TRX under cyclooxygenase-2 pathway (COX-2) evaluated by histopathological and morphometric analysis in segments of the duodenum. The tissues were submitted to H&E staining for morphometric and histopathological analysis after incubation with celecoxib. Salina (**A**); 5-FU (**B**); TRX-100 (**C**); CLX (**D**); TRX and CLX (**E**); height of villi (**F**); depth of the crypts (**G**); villus/crypt ratio (**H**). The histopathological issues are indicated by arrows. Inflammatory cells infiltration (red arrow), decreasing of intestinal villi (black arrow) and loss of intestinal crypt architecture (blue arrow). Values were expressed as mean ± SEM. For statistical analysis, one-way ANOVA followed by the Tukey test was used, where *p* < 0.05 vs. saline group, * *p* < 0.05 vs. 5-FU group, ** *p* < 0.05 vs. TRX group.

The morphometric analysis of the duodenum (Figure 7) showed that 5-FU (Figure 7B,F) promoted shortening of the villi (128.20 ± 10.76) and decreased the villus/crypt ratio (1.91 ± 0.16) when compared with the villus height (324.20 ± 15.04) and villus/crypt ratio (4.85 ± 0.29) observed in the Saline group (*p* < 0.05, Figure 7A). The shortening of villi induced by 5-FU was shown to be significantly prevented (*p* < 0.05) by treatment with 100 mg/kg TRX (183.90 ± 8.62, Figure 7C), 7.5 mg/kg celecoxib (CLX) (229.40 ± 7.5, Figure 7D) or a combination of TRX and CLX (291.00 ± 11.97, Figure 7E). Similarly, treatment with TRX or CLX alone or in combination reversed the effects of 5-FU and significantly increased (*p* < 0.05, Figure 7H) the villus/crypt ratio (2.98 ± 0.18, 3.55 ± 0.1, and 3.73 ± 0.23, respectively). Further, combination of TRX and CLX showed better prevention of villi shortening and villus/crypt ratio reduction than the TRX or CLX treatments alone (*p* < 0.05). For the deepening of the crypts, no significant differences were observed between the treatment groups.

Overall, the results (Figure 7A–E) showed that 5-FU promoted reduction of villus height, loss of villus and crypts architecture, edema, and increase in inflammatory infiltrate compared to that of saline treatment. Further, TRX and/or CLX treatment prevented the effects of 5-FU treatment.

### 2.9. Immunohistochemistry for the Detection of COX-2

**Figure 8:**
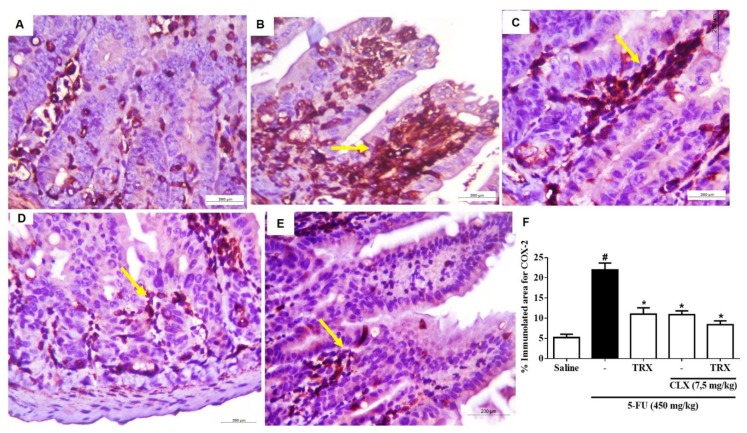
Immunohistochemistry analysis for COX-2 expression evaluation. Saline (**A**); 5-FU (**B**); TRX-100 (**C**); CLX (**D**); TRX and CLX (**E**); % immunolabelled for COX-2 (**F**). Values were expressed as mean ± SEM. For statistical analysis, one-way ANOVA followed by Tukey’s test was used, where # *p* < 0.05 vs. saline group, * *p* < 0.05 vs. group 5-FU.

We investigated the effects of TRX 100 mg/kg under COX-2 expression in the presence or absence of CLX during 5-FU-induced intestinal mucositis. The immunohistochemical analysis of duodenal mucosa showed higher expression of COX-2 (21.95 ± 1.70, *p* < 0.05) in 5-FU group (Figure 8B) than in Saline group (5.20 ± 0.81, *p* < 0.05) (Figure 8A,F). As shown in Figure 8C, treatment with TRX (100 mg/kg) significantly decreased the 5-FU induced COX-2 expression (11.01 ± 1.56, *p* < 0.05). Similarly, CLX treatment alone or in combination with TRX significantly decreased 5-FU-induced COX-2 expression (10.93 ± 1.56, *p* < 0.05, Figure 8D and 8.34 ± 0.92, *p* < 0.05, Figure 8E and 8F, respectively) in the intestinal mucosa of mice.

### 2.10. Molecular Docking

**Figure 9:**
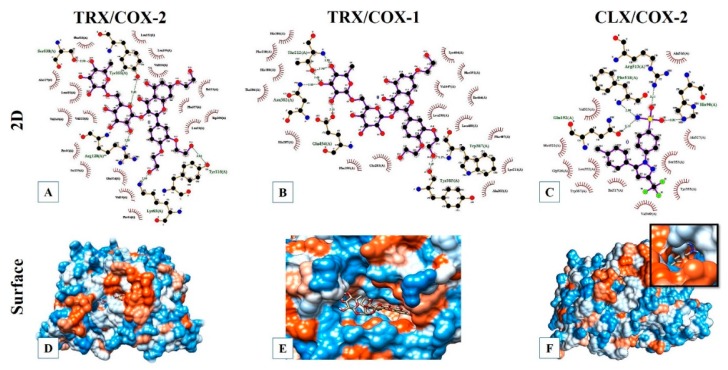
Molecular docking models of TRX or CLX with COX-1 and COX-2. (**A**), (**B**), and (**C**): Ligplot 2D diagrams showing details of hydrogen bond and hydrophobic interactions in complexes TRX/COX-2, TRX/COX-1, and CLX/COX-2, respectively. (**D**), (**E**), and (**F**): Surface docking poses of TRX/COX-2, TRX/COX-1, and CLX/COX-2, respectively.

Molecular docking analysis showed that the binding energy of TRX/COX-2 complex was −6.31 Kcal mol^−1^ and the inhibition constant was 23.64 µM. These values indicate that TRX has biological interaction with the target COX-2 enzyme. As shown in Figure 9A,D, TRX interacted with COX-2 at residues Ser530, Arg120, Lys83, Tyr115, and Tyr355 through hydrogen bonds and at residues Phe518, Val349, Ala527, Leu531, Val523, Pro86, Ser119, Glu524, Val89, Pro84, Trp100, Ile112, Leu93, Phe357, Leu359, Val116, and Leu352 through hydrophobic interactions. The molecular docking performed with TRX/COX-1 complex showed a binding energy of −5.63 Kcal mol^−1^ and an inhibition constant of 74.43 µM, showing possible bioactive activity of TRX against COX-1 enzyme. The interaction at the active site of COX-1 is possibly resulted because of hydrogen bonds at residues Asn382, Glu454, Thr212, Trp387, and Tyr385 (Figure 9B,E). The molecular docking results between CLX and COX-2 indicated a binding energy of −9.64 Kcal mol^−1^ and an inhibition constant of 85.56 µM. The interaction CLX/COX-2 also occurred due to hydrogen bonds at the residues Arg513, Phe518, His90, and Gln192, highlighting that the first amino acid forms an intense interaction between the atoms of NH1 and oxygen 01, which were separated with a distance of 2211 Å (Figure 9C,F).

## 3. Discussion

In this study, we demonstrated that TRX was able to prevent 5-FU-induced changes in the intestinal mucosa of mice. The prevention of intestinal mucositis was shown by different patterns of the intestinal mucosa cells and tissues, and biochemical characteristics, such as leukopenia, oxidative damage, neutrophilic recruitment, mastocytosis, goblet cell depletion, and alterations in the histological and morphometric data of the intestine.

For the current experimental evaluation, a segment of the duodenum was used. Previous analysis showed no difference between the duodenum and jejunum-ileum. The use of the duodenum instead of the jejunum-ileum was considered to avoid any microbial contamination and misinterpretation of the histological data.

Progressive weight loss is a typical characteristic of functional intestinal mucositis, usually caused by 5-FU chemotherapy, which culminates in decreased food intake and absorptive capacity [29]. In the present study, the body weight analysis showed that 5-FU treatment reduced the body mass of mice, which was irreversible by TRX during the 4 days of experimental protocol. Flavonoids have shown efficacy in treating intestinal mucositis and reverse weight loss [29,30]. However, the results are not consistent among the studies involving chemoprevention treatments. Studies performed by Cheah et al. [31] and Cechinel-Zanchett et al. [32], for example, demonstrated that flavonoids could not prevent the progressive weight loss induced by chemotherapy, even though their efficacy was shown in reversing histological changes, oxidative stress, and inflammatory process induced during mucositis. Weight loss is a side effect caused by the activation of inflammatory responses followed by gastrointestinal dysfunction [33]. The findings from our study corroborate with those of other studies, since TRX administration prevents morphophysiological changes in mucositis through mechanisms other than weight loss.

Several studies have described the histopathological and morphometric alterations promoted by 5-FU, such as reduction and vacuolization of intestinal villi, crypt necrosis, inflammatory cell infiltration, edema, loss of cell architecture, and decrease in villus/crypt ratio [34,35,36,37,38,39]. Our findings are consistent with those presented in these studies. It is during the ulceration phase where atrophic changes are visible including the lesion of the intestinal epithelium, a characteristic of mucositis [7,11,40].

By preventing and/or attenuating most of the changes in the intestinal mucosa induced by chemotherapy, TRX leads to the restoration of epithelial integrity, acceleration of the mucositis healing process, thus prevents ulcerative phase exacerbation and further deterioration. This process in the mucosa tissue is the reason for bacteremia and sepsis, which is one of the fundamental factors in worsening the prognosis of mucositis.

Concomitant with the restoration of epithelial integrity, the restoration of white blood cell count to baseline level is encouraging for the treatment and cure of mucositis. The leukopenia is another adverse effect of 5-FU chemotherapy treatment [41,42]. We found that TRX at doses of 100 and 150 mg/kg attenuated 5-FU-induced leukopenia. These findings indicate that TRX has a central role in the chemoprevention of complications associated with the pathophysiology of mucositis. Reversal of leukopenia helps in maintaining epithelial barrier integrity and basal leukocyte levels, which further protect the homeostatic microenvironment from microorganisms or inflammatory processes.

The neutrophils of the immune system constitute the first line of cellular defense. However, neutrophil over-activation results in the release of toxic mediators, such as myeloperoxidase (MPO), which promotes inflammatory processes as well as tissue damage [43,44,45,46,47]. In the present study, it was found that TRX (100 mg/kg) prevented 5-FU induced MPO release. The anti-inflammatory effect of TRX may be attributed to decreased MPO activity and consequently attenuation of neutrophil infiltration.

Many physiological mechanisms have been proposed to explain the anti-inflammatory action of flavonoids. Among these, the ability of flavonoids to inhibit neutrophil degranulation, modulation of immune cells involved in inflammation, such as natural killer cells, lymphocytes, macrophages and mast cells, are a few [48,49,50]. The mast cells have a critical role in regulating the innate and acquired immunity associated with the inflammation process [51,52]. The mast cell count in the duodenal segment of mucositis mice indicated that administration of TRX decreased the number of degranulated and granulated mast cells that were induced by 5-FU treatment. Another study has shown that TRX is capable of reversing mastocytosis [18]. According to Kheirollahi et al. [53], flavonoids reverse tissue damage by preventing neutrophil and mast cell degranulation, which corroborates with the findings obtained in this study.

In addition to the intestinal damage, chemotherapeutic agents may also compromise the mucosal protection layer by decreasing mucin, thereby increasing the exposure of the intestinal epithelium to harmful agents [54]. Based on the number of goblet cells in the duodenal segment of mice, it was concluded that TRX (100 mg/kg) prevents 5-FU-induced goblet cell depletion. The goblet cells are responsible for lining the intestinal epithelium with a protective mucus layer [55] and, thus, by preventing the goblet cell depletion, TRX may be responsible for the prevention of epithelial barrier damage.

The inflammatory processes, such as intestinal mucositis, results in an imbalance between the pro-oxidant and antioxidant systems causing oxidative stress. It is characterized by the production of reactive oxygen (ROS) and nitrogen (RNS) species, and free radicals. These reactive species interfere with the regular physiological process, which further causes structural and/or functional damage [56]. As shown by our study, TRX treatment (100 mg/kg) reverses the 5-FU-induced increase of MDA levels and prevents GSH consumption in mice. The antioxidant effect of TRX shown in the present study has also been reported by several published studies in different experimental models [17,18,19,21,57,58,59,60,61].

The wide range of therapeutic effects of flavonoids in the treatment of digestive tract disorders is attributed to their antioxidant properties [50]. Further, recent research has revealed that the gastroprotective action of flavonoids is caused by inhibiting cAMP, cyclooxygenase, and protein phosphorylation [49]. The molecular docking and histopathological evaluation used to investigate the interaction of TRX or CLX with COX-1 and COX-2 enzymes, found that TRX had a higher affinity towards COX-2 than COX-1.

Although cyclooxygenase enzymes have a similarity of about 60% in their structures, COX-2 shows conformational variations in the active site pocket, which may contribute to the differences in the sensitivity and affinity between COX-1 and COX-2 while interacting with ligands, such as TRX. Thus, COX-2 has a larger and more flexible substrate binding channel, which possibly justifies the higher affinity of TRX for COX-2 than COX-1 [62,63,64]. It was also shown that the binding sites in CLX/COX-2 complexes are distinct compared to those in the TRX/COX-2 complex. These computational evaluation results corroborate and support the in vivo experimental results performed using the histological, morphometric, and immunohistochemical analyses evaluating COX-2 expression pattern.

The histological analysis showed that not only TRX (100 mg/kg) but also CLX reverses the 5-FU-induced effects, as indicated by increase in villi height and villus/crypt ratio. The treatment with both TRX and CLX in combination showed better results in morphometric changes than with TRX (100 mg/kg) or CLX treatment alone. The immunohistochemical analysis showed a decrease in the proportion of mucosal cells stained with COX-2 antibody upon treatment with TRX or CLX alone or in combination.

The COX-2 plays a key role in inflammation through the release of arachidonic acid, and biosynthesis of eicosanoids, such as prostaglandins involved in various immune and inflammatory responses. Several studies have demonstrated the harmful effect of radiotherapeutic and chemotherapeutic agents, which result in COX-2 overexpression in intestinal mucositis models [48,65,66,67,68]. Hence, the anti-inflammatory effect of TRX on intestinal mucositis can be attributed to its binding and interaction with COX-2 and mediation of COX-2 pathway.

**Figure 10:**
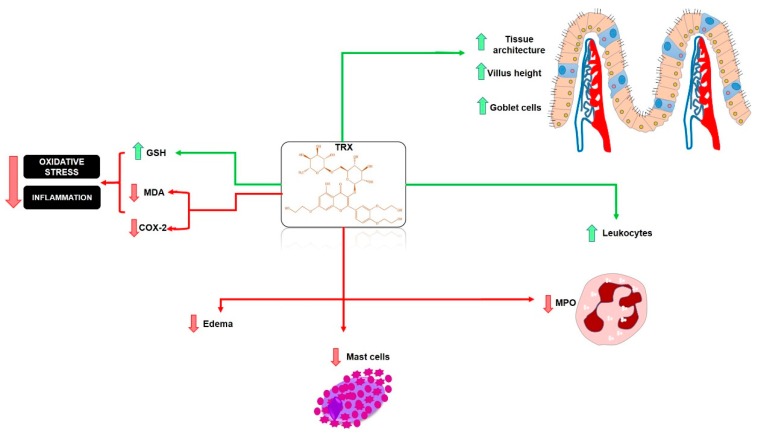
Hypothetical chemopreventive effect of TRX in intestinal mucositis induced by 5-FU. TRX prevented intestinal inflammation by inhibiting MDA, MPO, COX-2, ROS, mastocytosis, and inhibition of leukopenia. TRX also stimulated increased villi and increased levels of the antioxidant GSH. TRX: troxerutin; ROS: reactive oxygen species; COX-2: cyclooxygenase 2; MDA: malondialdehyde; MPO: myeloperoxidase; GSH: reduced glutathione. Green arrows (stimulate/increase), red arrows (inhibit).

The use of flavonoids in inflammatory diseases, especially mucositis is a current reality and a future promise for the development of effective chemopreventive drugs. This can be done by including the flavonoids in the treatment cycles of chemotherapy, such as 5-FU therapy, which causes intestinal injuries and mucositis. A hypothetical model showing the chemopreventive action of the flavonoid TRX on intestinal mucositis is shown in Figure 10.

## 4. Materials and Methods

### 4.1. TRX Synthesis

The rutin used in TRX semi-synthesis was provided by the Technological Development Park (PADETEC). The synthesis of TRX was performed according to a previously described methods with a few modifications [69,70]. It was synthesized in a 10 mL 3-mouth flask equipped with a magnetic stirrer and connected to ethylene oxide cylinder. First, 0.1 mL of 1N NaOH solution (0.1 mmol), a suspension of 0.61 g (1 mmol) of rutin and 1.5 mL of water were mixed and heated to 80–85 °C for one hour. Then, 0.2 g of ethylene oxide (4.5 mmol) was added to the mixture over a period of 6 h at a flow rate of 0.3 mL·min^−1^. When the pH of the mixture reached 9.5, the flask was depressurized, cooled, and the pH was adjusted to 4.5 with hydrochloric acid solution (1:2). Then, the mixture was filtered through a celite pad, concentrated on a rotary vacuum evaporator to a syrupy residue. The residue was stirred with 6 mL of warm ethyl acetate. The residue was separated from the acetate and then washed three times until a yellow powder was obtained. The powder was filtered on Buchner Funnel and dried in a vacuum oven at 70 °C yielding 0.67 g of hydroxyethylrutosides.

### 4.2. TRX Characterization

Chromatographic analysis of TRX was performed using a Shimadzu LC-10AD chromatography pump equipped with a UV detector. The wavelength and temperature was set at 255 nm and 25 ± 2 °C, respectively. The methodology proposed by Satinsky et al. [26] was used with minor modifications. The isocratic mobile phase solution consisting of acetonitrile/acetic acid (30:70 v.v^−1^) with pH adjusted to 3 was used with a flow rate of 1.0 mL·min^−1^.

The ^13^C NMR and ^1^H NMR data were obtained using the Fourier transform Bruker Avance-DRX 500 spectrometer (San Diego, CA, USA), equipped with an inverse detection probe operating at a frequency of 125 MHz (^13^C) and 499.9 MHz (^1^H). Twenty milligrams of TRX was dissolved in 0.6 mL of DMSO and the analysis was performed in 5 mm tubes. Chemical shifts (δ) are expressed in ppm.

### 4.3. Drugs and Reagents

Two drugs were used for mucositis induction and treatment, respectively: 5-FU (FauldFluor^®^, Libbs, Sao Paulo, Brazil) celecoxib (CLX-Celebra^®^, Pfizer, Sao Paulo, Brazil). Troxerutin was dissolved with physiological solution (0.9% NaCl) before use. All drugs and reagents were prepared immediately before use.

### 4.4. Animals

The animals were obtained from the Department of Surgery of the Federal University of Ceara (UFC). The male Swiss mice (25–30 g) were housed in polypropylene cages, lined with wood, in a controlled environment with a temperature of 23 ± 2 °C, in a cycle of 12 h light/12 h dark, with free access to water and standard feed. The procedures and experimental protocols were approved by the Ethics Committee on Animal Use of the Federal University of Ceara (CEUA-UFC) under number 2413051018.

### 4.5. Experimental Protocol of 5-FU-Induced Intestinal Mucositis

**Figure 11:**
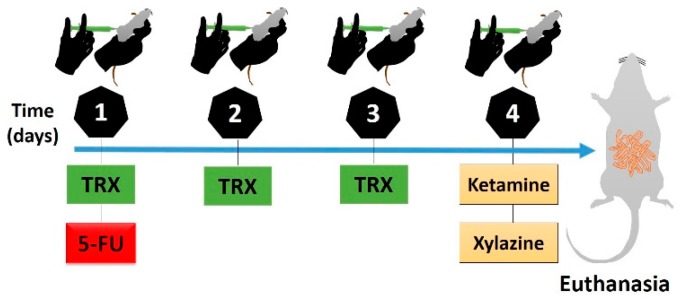
Scheme of induction and treatment of intestinal mucositis. TRX: troxerutin (day 1: 50 mg/kg; day 2: 100 mg/kg; day 3: 10 mg/kg); 5-FU: 5-Fluorouracil (day 1: 450 mg/kg, one hour after TRX); ketamine and xylazine (day 4: 270 and 15 mg/kg, respectively).

The experimental model of intestinal mucositis in Swiss mice was induced as described by Soares et al. [35]. 5-FU (450 mg/kg) was administered intraperitoneally (i.p) as a single dose on the first day of the experimental protocol. To evaluate the effective dose of TRX against 5-FU-induced morphological changes, 50, 100, and 150 mg/kg of TRX was administered orally on the first, second and third day respectively. First dose of TRX was administered 1 h before 5-FU injection, whereas, second and third doses were administered 24 and 48 h after 5-FU injection, respectively. On the fourth day of the experimental protocol, the animals were euthanized by anesthetic overdose of ketamine and xylazine (270 mg/kg and 15 mg/kg, respectively). Blood and intestinal samples were collected. The induction and treatment regimen is shown in Figure 11. The body weight of mice was assessed daily prior to administration of the administered treatment to confirm the experimental model of 5-FU induced intestinal mucositis. The TRX doses considered in the current study are in accordance with those in previous published studies [19,20,21,22,61].

To investigate the role of cyclooxygenase 2 (COX-2) enzyme during TRX treatment of 5-FU-induced intestinal mucositis, COX-2 was blocked by an intraperitoneal (i.p) injection of 7.5 mg/kg of CLX. Starting from the effective dose of TRX (100 mg/kg) for the treatment of intestinal mucositis, a 5-FU mucositis induction protocol was initiated, similar to the first investigation. For this, the mice were divided into three treatment groups, i.e., TRX-100 (100 mg/kg orally), CLX (7.5 mg/kg, i.p) and TRX + CLX (TRX: 100 mg/kg orally and CLX: 7.5 mg/kg, i.p), and two control groups, Saline and 5-FU. Overall, during the current study, mice were randomly divided to the following groups (n = 6 in each group): Saline (0.9% NaCl), 5-FU (450 mg/kg of 5-FU + 0.9% NaCl), TRX-50 (5-FU + 50 mg/kg TRX), TRX-100 (5-FU + 100 mg/kg TRX), TRX-150 (5-FU + 150 mg/kg TRX), CLX (5-FU + 7.5 mg/kg of CLX) and TRX + CLX (5-FU + 100 mg/kg TRX + 7.5 mg/kg CLX).

### 4.6. White Blood Cell Count

The mice were anesthetized with a combination of anesthetics (xylazine 10 mg/kg and ketamine 80 mg/kg) and a peripheral blood sample was collected from the ocular artery and diluted in Turk fluid at a ratio of 20 μL blood to 380 μL solution. Total leukocytes were counted using a Neubauer chamber [71] and the results were expressed as a total number of leukocytes per mm^3^ of blood.

### 4.7. Histopathological and Morphometric Analysis

After euthanasia, intestinal samples were collected and fixed in 10% formaldehyde for histopathological and morphometric analysis [35,72]. These samples were embedded in paraffin, sectioned at 4 μm and stained with hematoxylin and eosin (H & E). A blinded and randomized histopathological analysis was performed by an experienced histopathologist to assess the severity of mucositis using a scoring system [73]. Tissues ranged from 0 (no lesion/normal histological findings) to 3 (maximal grade lesion), indicating shortened villi vacuolated cells, crypt necrosis, intense inflammatory cell infiltration, vacuolization and edema in the mucous layer and muscle layer with edema, vacuolization and neutrophilic infiltrate. The effective dose of TRX for the treatment of intestinal mucositis was determined based on the histological analysis, leukocyte count and weight measurement.

### 4.8. Myeloperoxidase Assay (MPO)

MPO activity was determined by the technique described by Bradley et al. [74]. Briefly, samples from the duodenal segment (50–100 mg) corresponding to the animals of the Saline group, TRX 100 mg/kg and 5-FU (50–100 mg) were homogenized in 1 mL potassium buffer containing 0.5% hexadecyltrimethylammonium bromide (HTAB), then centrifuged (4000 rpm, 7 min, 4 °C). MPO activity was analyzed by measuring absorbance at 450 nm using diisocyanate dihydrochloride and 1% hydrogen peroxide in the resuspended pellet. The results were recorded as MPO units per mg of tissue.

### 4.9. Measurement of GSH and MDA Levels

The duodenal segment samples obtained from the animals of the Saline, TRX-100 and 5-FU groups were homogenized in cold EDTA or KCl (1:9, v/p) to prepare a 10% homogenate suspension for the estimation of GSH and MDA levels. The GSH levels were estimated according to method described by Sedlak and Lindsay [75], with minor modifications. Aliquots (400 μL) of homogenate tissue were mixed with 320 μL distilled water and 80 μL trichloroacetic acid (50%, w/v) and centrifuged at 3000 rpm for 15 min. The supernatant (400 μL) was mixed with 800 μL Tris buffer (0.4 M, pH 8.9), followed by addition of 5,5-dithiobis (2-nitrobenzoic acid) (DTNB; 0.01 M). The GSH absorbance was read at 405 nm and its concentration was expressed in μg/mg tissue. On the other hand, lipid peroxidation was determined by assessing the level of thiobarbituric acid reactive substances (TBARS) measured as MDA [76]. The homogenates were incubated at 37 °C for 1 h and added to 400 μL of 35% perchloric acid. The mixture was centrifuged (5000 rpm, 10 min at 4 °C) and 400 µL of 0.6% thiobarbituric acid was added to the supernatant, followed by incubation at 98 °C for 1 h. After cooling, the MDA absorbance was read at 532 nm and its concentration was expressed as nmol/mg tissue.

### 4.10. Intestinal Mucosa Cell Count: Goblet and Mast Cells

To enable the identification and quantification of mast cells and mucus-secreting cells (goblet), the paraffin blocks with samples from the duodenal segment, corresponding to the Saline, TRX-100 and 5-FU groups were selected for toluidine blue staining, according to Michalany et al. [77] and periodic acid Schiff (PAS) according to SANO et al. [78]. Stains were performed after de-paraffinization of the slide with xylol, followed by hydration with absolute alcohol and a series of 90%, 80%, 70% alcohol dilutions. Then, the slides were washed with distilled water, stained with toluidine blue for 8 min, washed and dried. For PAS, the slides were incubated in periodic acid, Schiff watering and Carazzi hematoxylin dyes for 1, 10, and 10 min, respectively, followed by successive washing with distilled water, as recommended by EasyPath^®^. For the counting of mast and goblet cells present in the slides, with the aid of an optical microscope to the image acquisition system (LEICA Wetzlar, HE, Germany), digital images were captured for subsequent counting of at least 10 fields, with the aid of ImageJ^®^ software. Results represent the average of 10 fields from each group.

### 4.11. Immunohistochemistry for the Detection of COX-2

Duodenal sections were deparaffinized with oven insertion (60 °C) and three cycles of xylol immersion for 5 min each. Then, the sections were rehydrated in decreasing alcohol concentrations (100%, 90%, 80%, and 70%). The histological sections were then washed with distilled water for 10 min and the antigenic recovery in citrate buffer (pH 7.0, DAKO^®^, Sao Paulo, Brazil) was carried out for 20 min in the water bath (95 °C). The slides were then washed with phosphate-buffered saline solution (PBS) for 5 min at room temperature. Following, endogenous peroxidase blockade with 3% hydrogen peroxide solution (H_2_O_2_) was performed for 30 min. The sections were then incubated overnight with goat anti-COX-2 primary antibody (SantaCruz^®^, Dallas, TX, USA), diluted in antibody diluent (1:100) for 60 min. After the slides were washed with PBS and incubated with rabbit IgG (GBI Labs^®^, Bothell, WA, USA) secondary antibody diluted (1:400) for 30 min. For revelation, the sections were incubated with the streptavidin conjugated peroxidase complex (ABC complex) for 30 min and chromogen 3,30diaminobenzidine peroxide, DAB (DAKO^®^, São Paulo, Brazil), followed by counterstaining with hematoxylin (DAKO^®^, Sao Paulo, Brazil), for 10 min. Negative controls were processed simultaneously as described above, with the primary antibody being replaced for antibody diluent. The procedures were performed in an automated manner using Autostainer Plus (DAKO^®^, Sao Paulo, Brazil). For COX-2 immunostaining images, quantification was performed by measuring the % immunolabelled area with the aid of Adobe Photoshop10. All images were captured with the aid of an optical microscope to the image acquisition system (LEICA, Wetzlar, HE, Germany).

### 4.12. Molecular Docking and Determination of TRX Binding Sites

The 3D structures of COX-1 and COX-2 enzyme targets were obtained from the PDB protein database (Protein Data Bank, 2019). Molecular docking calculations were performed using the Autodock 4.2^®^ program [79,80,81]. Proteins and ligands were prepared for molecular docking using the Autodock Tools (ADT) version 1.5.6 program. The receptor was considered rigid while each ligand was considered flexible. The Lamarckian Genetic Algorithm (LGA) with global search and pseudo-Solis and Wets with local search methods were used in molecular docking, and 100 independent runs were performed for each simulation [82]. The remaining docking parameters were set to default values. Molecular docking analyses were focused on the low energy clusters, and the conformation with lowest energy combined with visual inspection was chosen for detailed analysis.

### 4.13. Statistical Analysis

Results of the data with parametric distribution are expressed as mean ± standard error of the mean (SEM) and of the non-parametric distribution (e.g., histological scores) are expressed as the median. Data normality were analyzed using the Shapiro-Wilk test.

The results with a parametric distribution were analyzed by Analysis of Variance (ANOVA) followed by post hoc test Tuckey through the program GraphPad Prism version 6.0 (GraphPad Software Inc., La Jolla, CA, USA). The data obtained from non-parametric distribution were analyzed using Kruskal–Wallis test followed by Dunn’s (multiple comparisons). Values of *p*-value < 0.05 were considered statistically significant.

## 5. Conclusions

In summary, TRX prevented the functional and inflammatory changes induced by 5-FU in intestinal mucositis. These chemopreventive effects were shown by reversal of leukopenia, histopathological and morphometric changes, oxidative damage, neutrophilic infiltrate, mastocytosis, and goblet cell depletion. The effects of TRX could be via the COX-2 pathway, which is supported by decreased COX-2 immunostaining and higher binding affinity between TRX and COX-2 as shown by molecular docking analysis. However, further studies may be required to elucidate the underlying molecular mechanisms of TRX on the treatment of chemotherapy-induced intestinal mucositis as well as its effect in the presence of proinflammatory cytokines.

## Figures and Tables

**Table 1:** ^1^H NMR and ^13^C NMR data for troxerutin (DMSO-d_6_, δ, multiplicity).

^1^H NMR		^13^C NMR	
Troxerutin	δ (ppm)	Troxerutin	δ (ppm)
5-OH	12.5 (s)	4-C	177.9
2′-Ar	7.8 (s)	7-Ar	165.1
6′-Ar	7.7–7.6 (d)	9-C	161.3
5′-Ar	7.1 (d)	5-Ar	157.0
8-Ar	6.7 (s)	2-C	156.9
6-Ar	6.3 (s)	4*′*-Ar	151.3
CH_2_CH_2_OH	5.4–5.3 (m)	3*′*-Ar	148.0
CH_2_CH_2_OH	4.1–4.0 (m)	3-C	134.1
CH_2_CH_2_OH	3.5–3.2 (m)	1*′*-Ar	123.0
6‴-CH_3_	0.9 (m)	6*′*-Ar	122.8
		5*′*-Ar	114.8
		2*′*-Ar	113.2
		10-Ar	105.5
		1″-C	101.7
		1*‴*-C	101.4
		6-Ar	98.8
		3*″*-C	76.8
		5*″*-C	74.6
		4*‴*-C	72.2
		3*‴*-C	71.0
		OCH_2_CH_2_OH	70.8
		5*‴*-C	68.7
		CH_2_OH	59.9
		CH_2_OH	59.7
		6*‴*-C	18.2

**Table 2:** Histopathological scores of mice subjected to 5-FU-induced intestinal mucositis and pretreated with TRX.

Groups	Scores
Saline	0 (0–1)
5-FU	3 (2–3) ^a^
TRX-50	2 (1–3)
TRX-100	1 (1–2) ^b^
TRX-150	3 (1–3)

Values were expressed as median, where **^a^** *p* < 0.05 vs. saline and **^b^** *p* < 0.05 vs. 5-FU (n = 6/group). The data was analyzed by the Kruskal–Wallis test followed by the Dunns multiple comparisons test.
